# C-terminal binding protein-2 promotes cell proliferation and migration in breast cancer via suppression of p16^INK4A^

**DOI:** 10.18632/oncotarget.15402

**Published:** 2017-02-16

**Authors:** Xiaojing Yang, Yi Sun, Hongling Li, Yuhui Shao, Depeng Zhao, Weiwei Yu, Jie Fu

**Affiliations:** ^1^ Department of Radiation Oncology, Shanghai Jiao Tong University Affiliated Sixth People's Hospital, Shanghai, 200233, China; ^2^ Department of Obstetrics, Shanghai First Maternity and Infant Hospital, Tongji University School of Medicine, Shanghai 200040, P.R. China

**Keywords:** CtBP2, p16^INK4A^, breast cancer, proliferation, migration

## Abstract

C-terminal binding protein-2 (CtBP2) enhances cancer proliferation and metastasis. The role and mechanism of CtBP2 in breast cancer remains to be elucidated. Western blot and immunochemistry were employed to evaluate the level of CtBP2 and p16^INK4A^ in breast cancer. Genetic manipulation was used to study the expression of p16^INK4A^ and its downstream genes regulated by CtBP2. Functional assays, including colony formation, wound healing, transwell invasion, anchorage-independent growth assay and a xenograft tumor model were used to determine the oncogenic role of CtBP2 in breast cancer progression. The expression of CtBP2 was increased in breast cancer tissues and cell lines. The expression of p16^INK4A^ were inversely correlated CtBP2 (*r*^2^ = 0.43, *P* < 0.01). The expression of both CtBP2 and p16^INK4A^ were significantly related to histological differentiation (*P* < 0.01 and *P* = 0.004, respectively) and metastasis (*P* = 0.046 and 0.047, respectively). The overall survival rate was lower in patients with increased CtBP2 expression and lower p16^INK4A^ expression. Knockdown of CtBP2 resulted in the activation of p16^INK4A^ and down–regulation of cell cycle regulators cyclin D, cyclin E and cyclin-dependent kinase 2 and 4. This down-regulation also led to a decreased transition of the G1-S phase in breast cancer cells. Moreover, gain-of-function experiments showed that CtBP2 suppressed p16^INK4A^ and matrix metalloproteinase-2, subsequently enhancing the migration in breast cancer. However, the silence of CtBP2 abrogated this effect. Collectively, these findings provide insight into the role CtBP2 plays in promoting proliferation and migration in breast cancer by the inhibition of p16^INK4A^.

## INTRODUCTION

Breast cancer is currently one of the most common malignancies, and the second leading cause of cancer death among women worldwide [1]. Despite the progress in combined modality therapies, the long-term outcome of patients with breast cancer is far from satisfactory [2]. This outcome is mainly attributed to the induction and progression of breast cancer. Several studies show that aberrant transcriptional activities of major oncogenes and tumor suppressor genes are involved in the tumorigenesis of breast cancer [3]. Although the identification and characterization of transcriptional co-activators and co-repressors further the understanding of regulation on oncogene transcription, the underlying mechanisms by which specific transcription factors play a role in breast cancer are still unclear.

Recently, C-terminal binding protein family proteins (CtBP1 and CtBP2), members of the co-repressors family, are reported to be involved in several essential cellular processes related to tumorigenesis [4]. CtBP2-mediated repression results in the inhibition of p16^INK4A^ [5], E-cadherin [6], PTEN [7, 8] and PERP (p53-effector related to pmp-22) [4] leading to the oncogenesis [9]. Clinically, the expression of CtBP2 is increased in patients with malignant cancers [10–14], and associated with a poorer prognosis.

p16^INK4A^ is a cyclin-dependent kinase (CDK) inhibitor that has multiple biological functions, including the inhibition of cell cycle progression [15, 16], the modulation of DNA damage–induced apoptosis [17], and the repression of migration [18, 19]. The protein expression of p16^INK4A^ is reduced in human primary tumors, including those of ESCC (esophageal squamous cell carcinoma) [14], urothelial cancer [20], ovarian cancer [21], non-small cell lung carcinoma, glioma and breast carcinoma [22]. p16^INK4A^ blocks the cell cycle progression by binding to either CDK4 or CDK6, and inhibiting the action of cyclin D [23–26]. In the presence of an imbalance between p16^INK4A^ and cyclin D, the p16^INK4A^ accelerates the G1-S phase checkpoint, resulting in abnormal cell growth and tumor development [27]. Down-regulation of p16^INK4A^ promotes migration in breast cancer because of an increased secretion level of matrix metalloproteinase-2 (MMP-2) [19]. In the present study, we investigated the detailed mechanisms by which CtBP2 contributes to the development of breast cancer and the predictive value of CtBP2 and associated pathways in the prognosis of breast cancer.

## RESULTS

### The expression of CtBP2 and p16^INK4A^ in breast cancer tissues and cells

**Figure 1:**
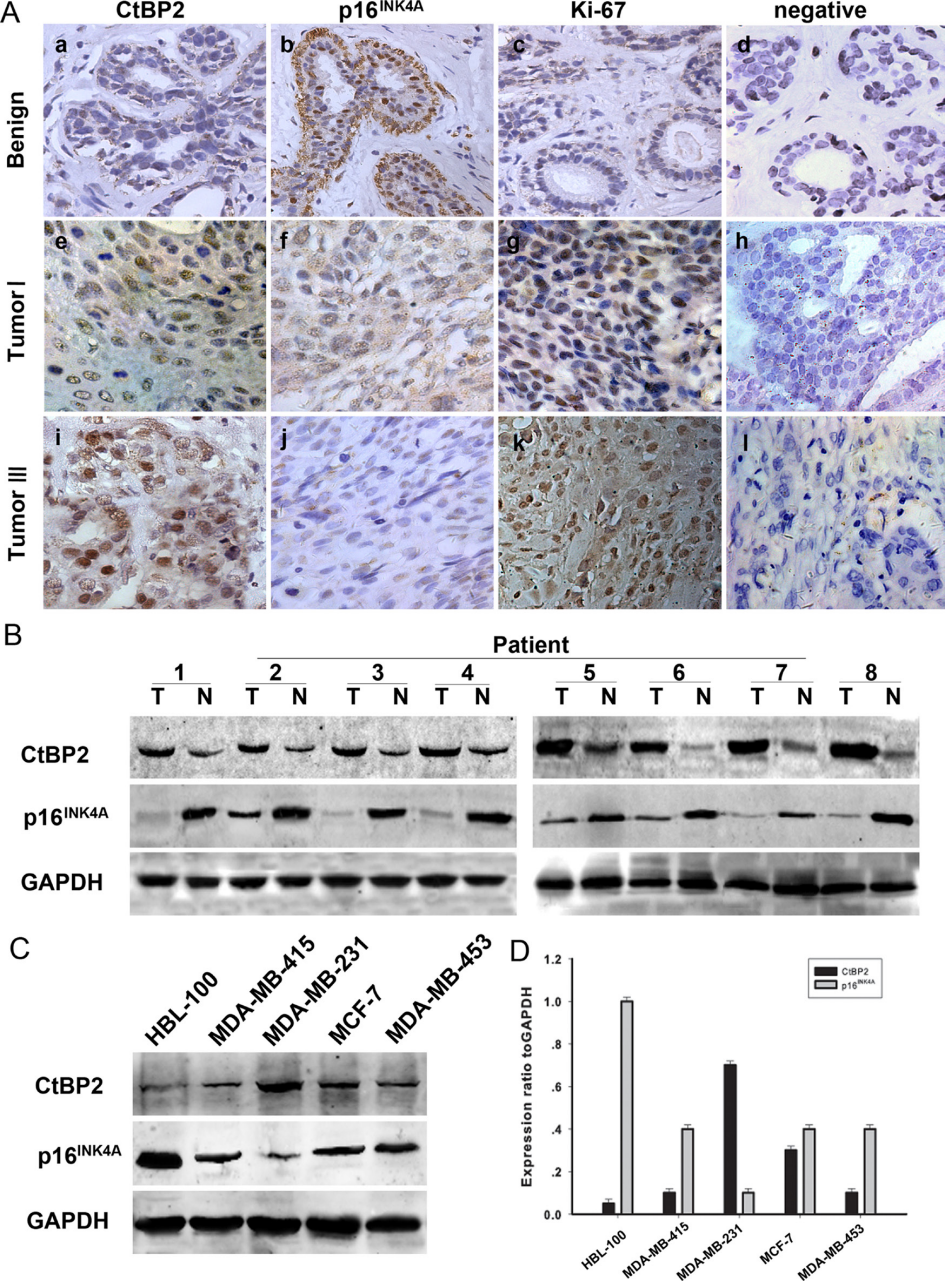
Expression of CtBP2 and p16^INK4A^ in human breast cancer (**A**) Paraffin-embedded tissue sections were stained with antibodies for CtBP2, p16^INK4A^ and Ki-67 and then counterstained with hematoxylin. Figure a–c, e–g Low CtBP2 and Ki-67 expression was observed in benign breast disease and breast carcinoma specimens (grade I), whereas p16^INK4A^ levels were low in the same specimens (SP×400). Figure i-k High levels of CtBP2 and Ki67 were observed in grade III tumor cells. In contrast, p16^INK4A^ expression was low. Figure d, h, and l show negative controls for the benign breast disease and the breast carcinoma specimens. (**B**) Expression of CtBP2 and p16^INK4A^ in eight representative paired samples of breast cancer and adjacent normal tissues. (**C**) Western blot analysis of endogenous CtBP2 and p16^INK4A^ in normal human breast epithelial cells HBL-100 and four human breast cancer cell lines (MDA-MB-415, MDA-MB-231, MCF-7 and MDA-MB-435). GAPDH was used as a loading control. (**D**) Quantification indicated the levels of CtBP2 and p16^INK4A^ in these cells. The experiments were repeated at least three times.

Immunohistochemical staining was used to determine the physiological and pathological interaction between CtBP2, p16^INK4A^ and the proliferation index Ki-67 in tissue samples from patients with both benign breast disease and breast cancer. The results are shown in Figure 1A and Table 1. Representative examples of reactivity for CtBP2, p16^INK4A^ and Ki-67 are shown in Figure 1A. CtBP2 and p16^INK4A^ were both expressed mainly in the nuclei. Our results showed that the immunoreactivity of CtBP2 and Ki-67 was weak in the nucleus of (Figure 1Aa, 1Ac) mammary epithelial and myoepithelial cells in tissue samples from benign breast disease. The expression of p16^INK4A^ was strong (Figure 1Ab); however, CtBP2 was strongly expressed in tissues from breast cancer samples and four breast cancer cell lines. Interestingly, the expression of CtBP2 was higher in MDA-MB-231 and MCF-7 cells when compared with other cell lines (Figure 1C, 1D). Moreover, we found that the level of p16^INK4A^ was inversely related to the level of CtBP2 in both breast carcinoma specimens and cell lines of breast cancer (Figure 1B).

**Table 1:** Association of CtBP2 and p16^INK4A^ expression with clinicopathological parameters in 80 breast cancer specimens

Parameters	Total	CtBP2 expression		*P*	p16^INK4A^ expression		*P*
		Low ≤ 0.61	High > 0.61		Low ≤ 0.37	High > 0.37	
Age (years)							
≤ 50	31	12	19	0.135	14	17	0.187
> 50	49	11	38		16	33	
Histological grade							
Well	14	12	2	0.000*	0	14	0.004*
Mod	41	9	32		17	24	
Poor	25	2	23		13	12	
Metastasis							
Positive	56	7	49	0.046*	28	28	0.047*
Negative	24	16	8		2	22	
Tumor size(cm)							
≤ 5	28	17	11	0.011*	17	11	0.043*
> 5	52	6	46		13	29	
Histology							
Ductal	65	17	48	0.117	23	42	0.316
others	15	6	9		7	8	
ER							
+	49	13	36	0.167	18	31	0.480
–	31	10	21		12	19	
PR							
+	47	12	35	0.183	20	27	0.511
–	33	11	22		10	23	
HER2 status							
+	26	11	15	0.103	14	12	0.157
–	54	12	42		16	38	

Statistical analyses were performed by the Pearson χ^2^ test.

*P < 0.05 is considered significant.

**Figure 2:**
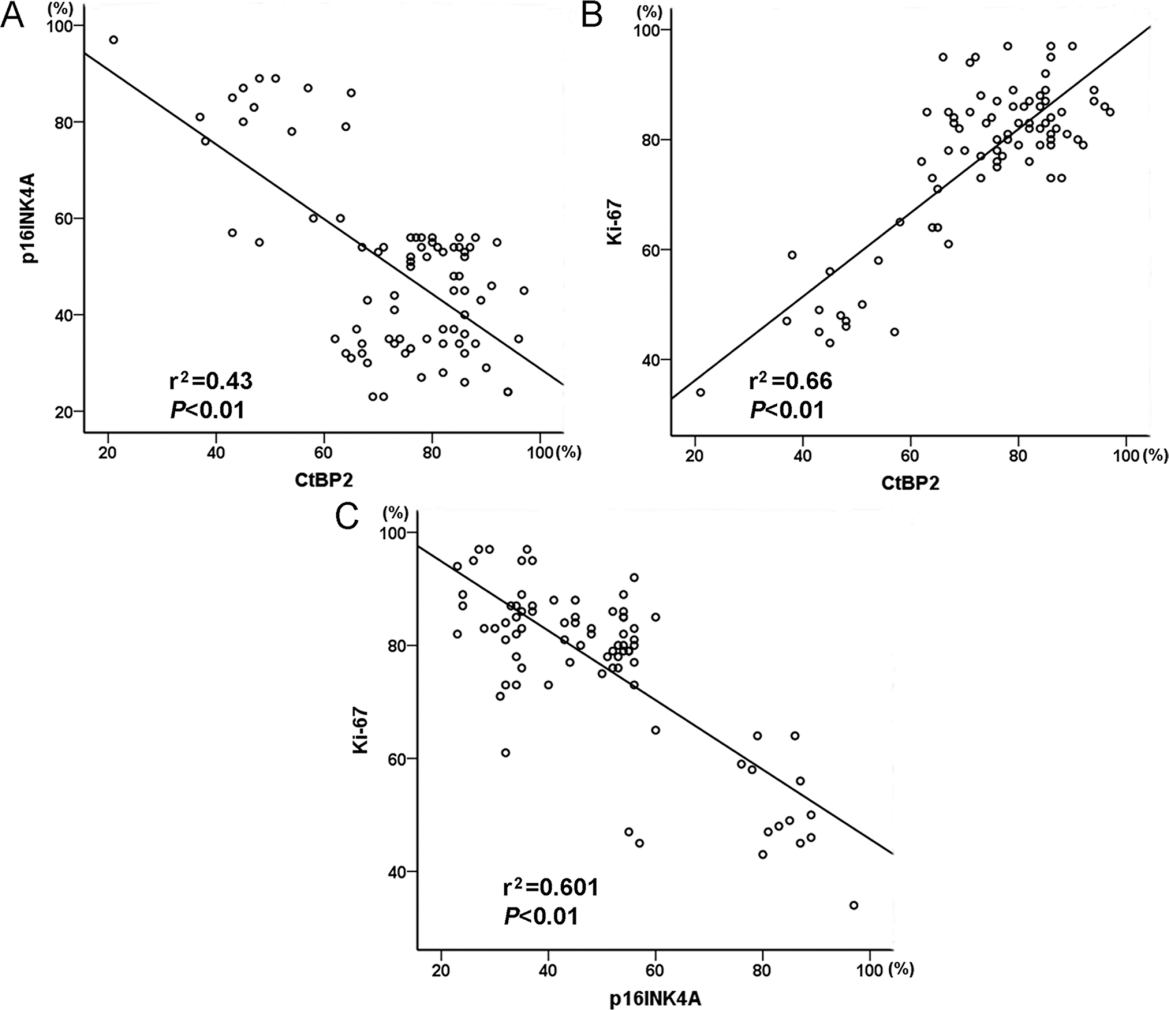
Graphic representation of relationship between CtBP2, p16^INK4A^ and Ki-67 expression in breast cancer (**A**) The relationship between CtBP2 and p16^INK4A^. (**B**) The relationship between CtBP2 and Ki-67. C The relationship between p16^INK4A^ and Ki-67.

The expression of CtBP2 was positively related to Ki-67 in breast cancer specimens (Figure 2). In addition, the proportion of p16^INK4A^-positive tumor cells was negatively correlated with the proportion of CtBP2-positive and Ki-67-positive tumor cells (Figure 2).

### Correlation between CtBP2, p16^INK4A^ expression and clinicopathological variables in breast cancer

As shown in Table 1, the level of CtBP2 was positively correlated with the histologic grade (*P* < 0.001), metastasis (*P* = 0.046) and tumor size (*P* = 0.011). However, CtBP2 expression was not related to the age, histology, estrogen receptors (ER), progesterone receptors (PR) or HER2 status in patients with breast cancer. In contrast, the level of p16^INK4A^ expression was inversely correlated with histologic grade (*P* = 0.004), metastasis (*P* = 0.047) and tumor size (*P* = 0.043), and no significant correlation was found between p16^INK4A^ expression and other variables.

### The expression of CtBP2 and p16^INK4A^ in relation to prognosis in patients with breast cancer

**Figure 3:**
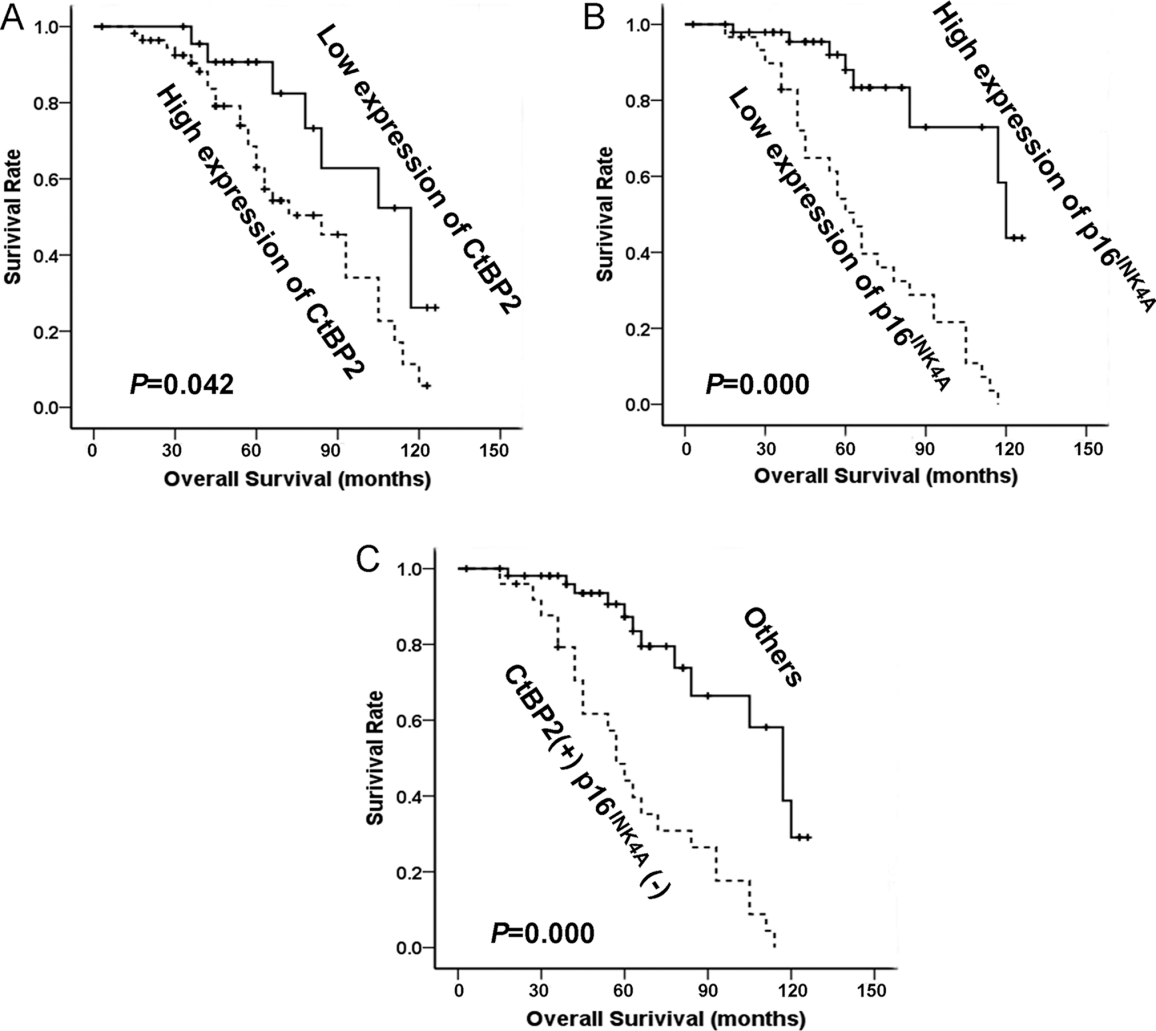
The relationship between CtBP2, p16^INK4A^ and patient survival (**A**) Based on mean CtBP2 percentages, patients were divided into high CtBP2 expressers (> 61.36%) and low CtBP2 expressers (≤ 61.36%). Patients in the high-expression CtBP2 group had significantly shorter overall survival. (**B**) Patients were also divided into two groups according to p16^INK4A^ expression both high expressers (> 37.14%) and low expressers (≤ 37.14%). Patients in the low-expression p16^INK4A^ group had significantly shorter overall survival. (**C**) Patients with CtBP2 (+)/p16^INK4A^ (−) phenotype (CtBP2 > 61.36% and p16^INK4A^ ≤ 37.14%) had the worst cumulative survival.

At the end of clinical follow-up, survival information was available for a total of 80 patients. The survival rate of patients with a high level of CtBP2 was significantly lower than that of patients with a low level of CtBP2 (31.2%, (18/57) and 78.3% (18/23), respectively), as shown in Table 2. Univariable analysis was performed to study the expression of CtBP2 and p16^INK4A^ in relation to survival status (Table 2). Kaplan–Meier analysis showed that increased expression of CtBP2 was significantly associated with shorter overall survival (*P* = 0.042, Figure 3A), whereas a high level of p16^INK4A^ was associated with longer overall survival (*P* < 0.001, Figure 3B). Patients with a high expression of CtBP2 and low expression of p16^INK4A^ had a poorer overall survival rate when compared to the other patients (*P* < 0.001, Figure 3C). The Cox's proportional hazards regression model demonstrated that expression level of CtBP2 and p16^INK4A^, histological grade, tumor size and metastases were independently predictive factors for an adverse prognosis in patients with breast cancer (Table 3).

**Table 2:** Survival status and clinicopathological parameters in 80 breast carcinomas specimens

	Total	Survival status		*P*	χ^2^
		Alive	Dead		
Age(years)					
≤ 50	31	13	18	0.818	0.192
> 50	49	23	26		
Histological grade					
Well	14	3	11	0.030*	6.997
Mod	41	17	24		
Poor	25	16	9		
Metastasis					
Positive	56	16	40	0.047*	1.810
Negative	24	20	4		
Tumor size(cm)					
≤ 5	28	20	8	0.031*	2.212
> 5	52	16	36		
ER					
+	49	20	29	0.132	1.033
–	31	16	15		
PR					
+	47	22	25	0.276	0.738
–	33	14	19		
CtBP2					
Low ≤ 0.61	23	18	5	0.029*	2.871
High > 0.61	57	18	39		
p16^INK4A^					
Low ≤ 0.37	30	4	26	0.002*	15.306
High > 0.37	50	32	18		

Statistical analyses were performed by the Pearson χ^2^ test.

*P < 0.05 is considered significant.

**Table 3:** Contribution of various potential prognostic factors to survival by Cox regression analysis in 80 breast carcinomas specimens

	Hazard ratio	95% CI	*P*
Age (years)	1.732	0.875~3.430	0.115
Histological grade	2.489	1.505~4.118	0.000*
Metastasis	2.168	1.702~4.070	0.048*
Tumor size (cm)	2.815	1.376~3.766	0.044*
ER	0.476	0.213~0.987	0.314
PR	0.737	0.318~1.521	0.389
CtBP2	2.397	1.077~5.333	0.032*
p16INK4A	0.161	0.070~0.375	0.000*

Statistical analyses were performed by the log-rank test.

*P < 0.05 is considered significant.

### The expression of CtBP2 and p16^INK4A^ was correlated to cell cycles in the MDA-MB-231 breast cancer cell line

**Figure 4:**
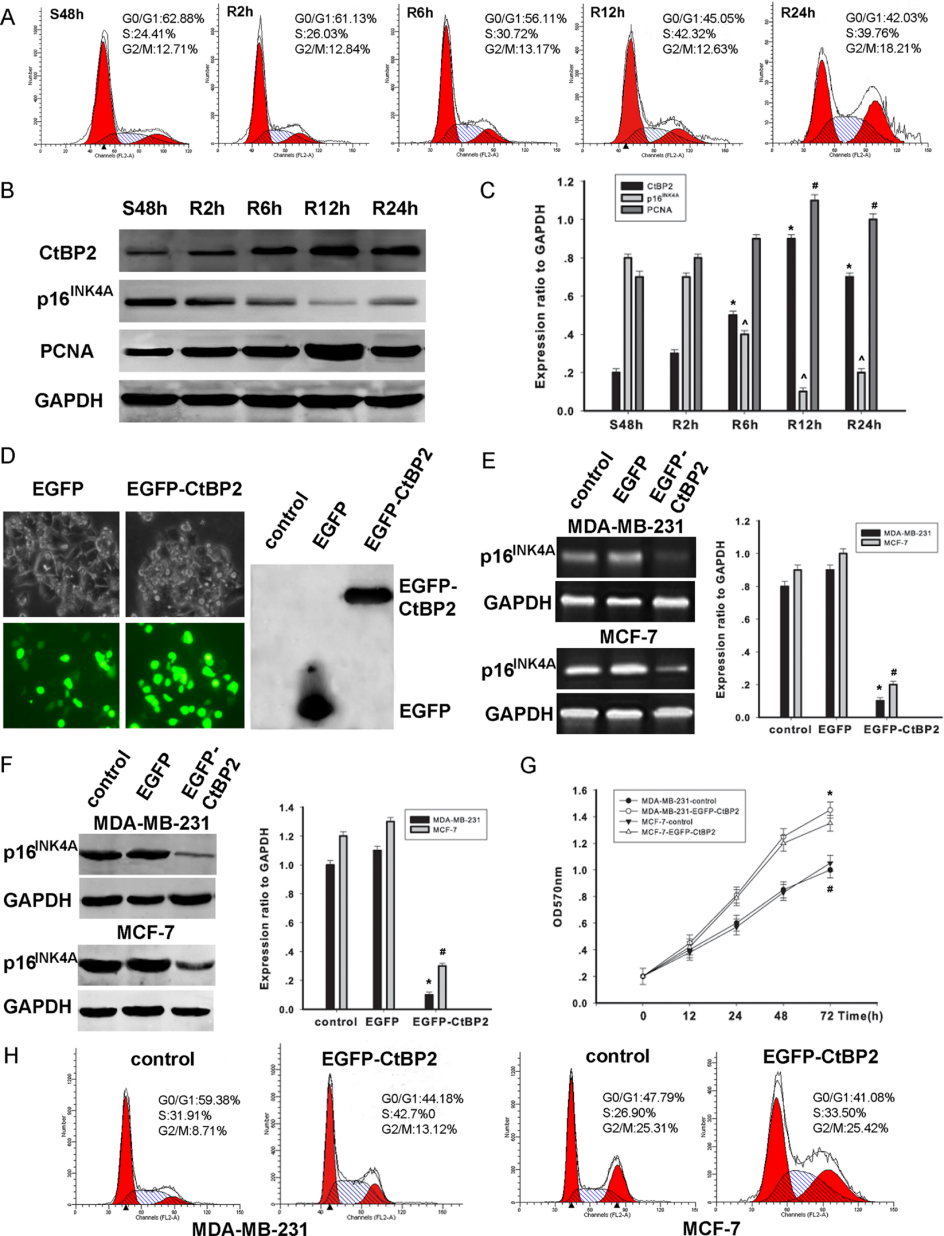
CtBP2 plays a proliferative role in breast cancer cells (**A**) Flow cytometry analysis of cell cycle progression in MDA-MB-231 cells. Cells that were synchronized at G1 progressed into the cell cycle 0, 2, 6, 12, and 24 h after serum stimulation. Finally, most of the cells entered S phase. (**B**) MDA-MB-231 cells were serum starved for 48 h (S48h). Upon serum stimulation, cell lysates were prepared and analyzed by Western blot using antibodies against CtBP2, p16^INK4A^, PCNA and GAPDH. GAPDH was used as a control for loading and protein integrity. (**C**) The bar graph demonstrates the ratio of CtBP2, p16^INK4A^ and PCNA proteins to GAPDH at each time point, as determined by densitometry. The data are represented as the mean ± SEM (*n* = 3, *^^#^*P* < 0.01, compared with control: S48 h). S: serum starvation; R: serum stimulation. (**D**) Light microscopy shows that pcDNA3.1-EGFP and pcDNA3.1-EGFP-CtBP2 are expressed in MDA-MB-231 cells. Proteins were analyzed by Western blot using an anti-EGFP antibody. (**E**) MDA-MB-231 and MCF-7 cells were transfected with pcDNA3.1-EGFP- CtBP2 or nothing (control). RT-PCR shows transcriptional levels of the p16^INK4A^ gene 48 h post-fection, and GAPDH was used as a loading control. The data are means ± SEM *^#^*P* < 0.01, compared with the control group. (**F**) MDA-MB-231 and MCF-7 cells were transfected with the pcDNA3.1-EGFP-CtBP2, or control, as indicated. Protein expression of p16^INK4A^ and GAPDH was analyzed by Western blot. Data are presented as means±SEM *^#^*P* < 0.01, compared with the control group. (**G**) MDA-MB-231 and MCF-7 cells were transfected with either pcDNA3.1-EGFP-CtBP2 or control. Cell growth of the transfected cells was assessed by the CCK-8 cell viability assay. The data are presented as the mean±standard error of three experiments. (**H**) Cell cycle analysis was performed by staining CtBP2 overexpressing MDA-MB-231 and MCF-7 cells with PI.

The correlation between the cell cycle stages and the expression levels of CtBP2 and p16^INK4A^ was further examined in MDA-MB-231 cells. After the synchronization of cell cycles at the G0/G1 phase by serum deprivation for 48 h, the cells were released and allowed to progress to the S phase by serum stimulus. The progress of the entire cell cycle was monitored by flow cytometry as the time indicated (Figure 4A). We found a time–dependent increase of CtBP2 expression (Figure 4B). In contrast, the p16^INK4A^ expression showed a trend of time–dependent decrease (Figure 4C). Collectively, our findings suggest that CtBP2 and p16^INK4A^ expression is related to the cell cycle. Our results are consistent with a previous study by Guan et al. [14].

### CtBP2 promotes proliferation in breast cancer cell lines

We further investigated the mechanisms by which CtBP2 stimulates proliferation in cell lines of MDA-MB-231 and MCF-7. The transfection of pcDNA3.1-EGFP and pcDNA3.1-EGFP-CtBP2 vectors in MDA-MB-231 cells was tested by light microscopy. In addition, CtBP2 protein was analyzed using Western blot (Figure 4D). The expression of p16^INK4A^ was significantly decreased in cells transfected with pcDNA3.1-EGFP-CtBP2 vectors when compared to cells transfected with pcDNA3.1-EGFP vectors (Figure 4E). Concomitantly, a decrease of p16^INK4A^ expression was detected (Figure 4F). After transfection, cell proliferation was evaluated by CCK-8 assay at indicated times (0, 12, 24, 48, and 72 h). The results demonstrate a remarkable increase of cell proliferation (Figure 4G). In addition, fluorescence activated cell sorter analysis of cell cycle distribution revealed that the number of cells in S phase was significantly increased in cells transfected with pcDNA3.1-EGFP-CtBP2 vectors (Figure 4H). Our findings suggest that CtBP2 promotes cell proliferation by inhibiting p16^INK4A^, resulting in a shorter transition of the cell cycle in breast cancer cells.

**Figure 5:**
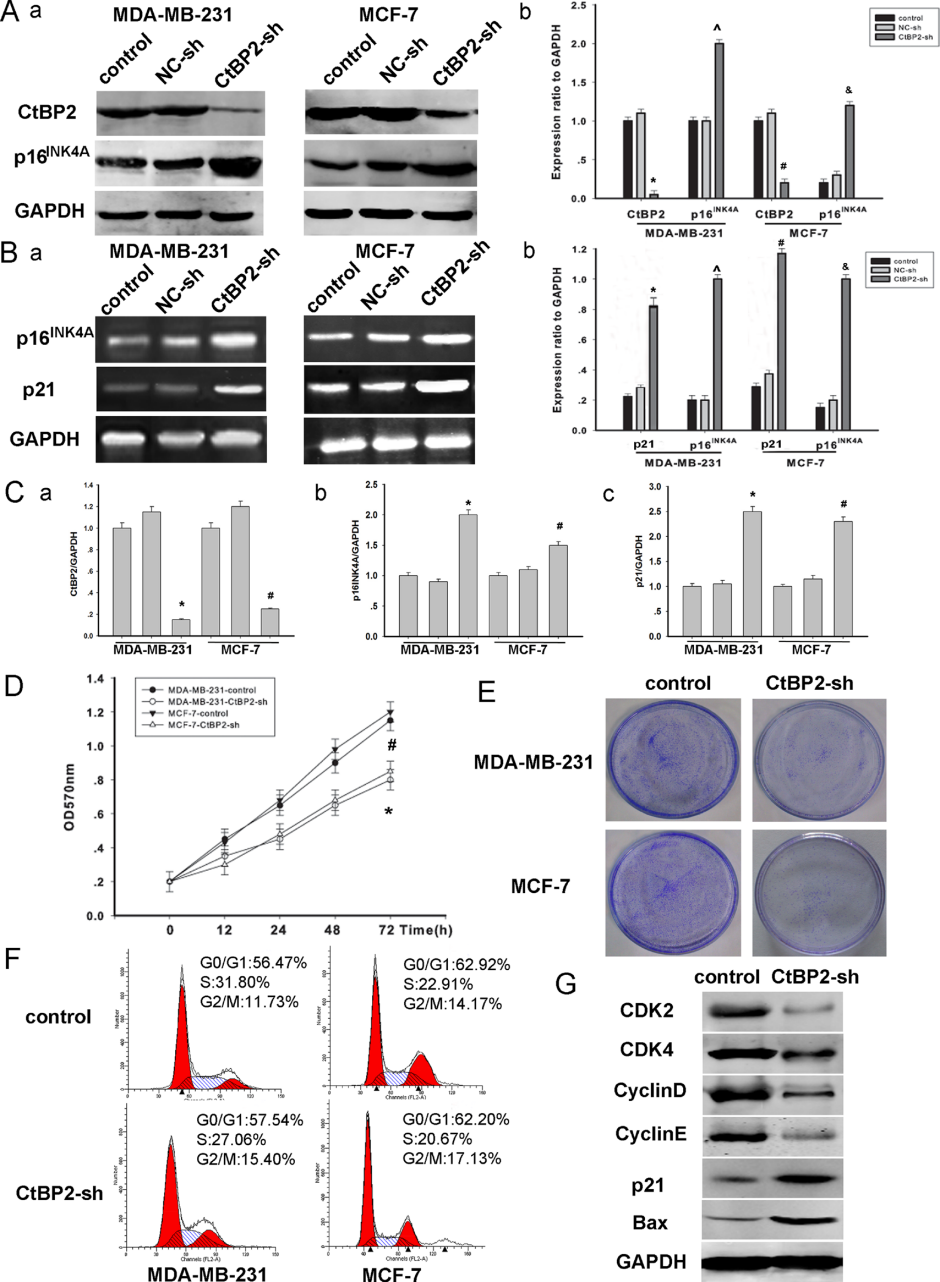
Knockdown of CtBP2 declines breast cancer cells proliferation (**A**) MDA-MB-231 and MCF-7 cells were transfected with shRNA targeting either CtBP2 or a scrambled sequence (control shRNA) as described above for 48 h. Western blot analysis of CtBP2, p16^INK4A^ and GAPDH were performed. (**B**) RT-PCR shows transcriptional levels of the p16^INK4A^ and p21 gene 48 hours post transfection and GAPDH was used as a loading control. The data are means ± SEM *^#^*P* < 0.01, compared with the control group. (**C**) Analysis of the expression of CtBP2, p16^INK4A^ and p21 by PT-qPCR. (**D**) *In vitro* cell growth was examined by cell proliferation assay at the indicated time. The data are means ± SEM (*n* = 3, *P < 0.05, compared with control cells). (**E**) Silencing endogenous CtBP2 inhibits cell growth as determined by colony formation assays. (**F**) 48 h post-transfection, cells transfected, as described above, were stained with PI for DNA content analysis by FACS. Details of the experiments are given in “Materials and Methods”. (**G**) A representative Western blot image showed the expression of CDK2, CDK4, Cyclin D, Cyclin E, p21 and BAX in control and CtBP2-shRNA treated cells, respectively. GAPDH was used as internal control.

To further investigate the CtBP2–p16^INK4A^ pathway described above, we established loss–of–function models (Figure 5). MDA-MB-231 and MCF-7 cells were transfected with either CtBP2-shRNA or control vectors for 48 h. The efficiency of transfection was assessed by Western blot (Figure 5A). We found that the expression of p16^INK4A^ was significantly increased after the transfection of CtBP2-shRNA vectors (Figure 5A, 5B, 5C). Moreover, CCK8 and colony formation assays revealed that the proliferation rate of CtBP2-shRNA positive cells was significantly slower (Figure 5D, 5E). Additionally, the analysis of cell cycle distribution revealed an accumulation of cells at the G0/G1 phase and a concomitant reduction of cells at S phase after the transfection of CtBP2-shRNA vectors (Figure 5F). The expression of several key cell cycle regulators, including CDK2, CDK4, CyclinD and CyclinE was decreased in CtBP2-shRNA positive cells (Figure 5G). We also measured the expression of p21 and Bax which are additional CtBP2-targeted pathways [4]. We found that the expression of p21 and Bax was increased in CtBP2-shRNA positive cells (Figure 5G).

### CtBP2 facilitates breast cancer cell migration and invasion

**Figure 6:**
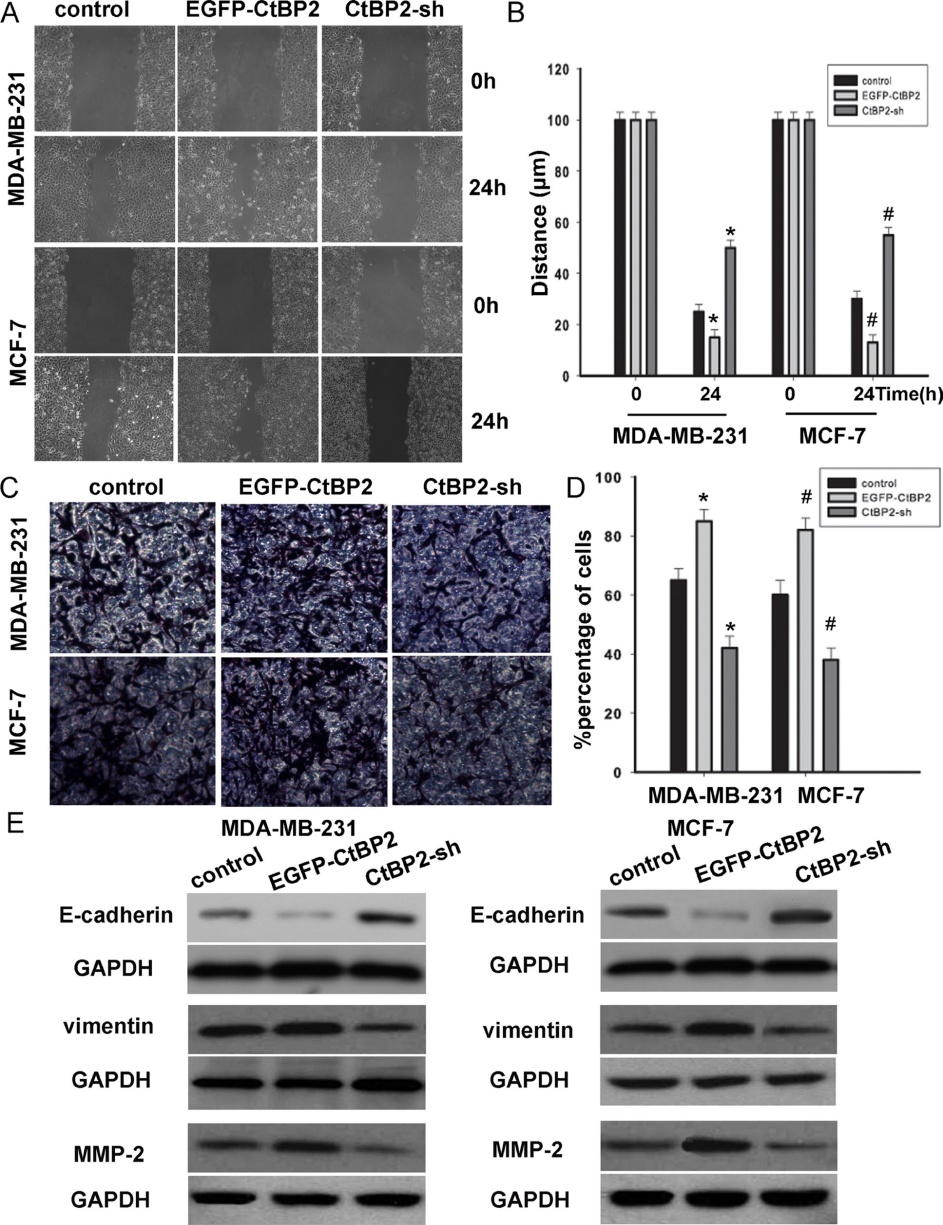
CtBP2 suppressed breast cancer cells migration and invasion (**A** and **C**) MDA-MB-231 and MCF-7 cells were transfected with or without EGFP-CtBP2 and CtBP2-shRNA as indicated. Wound-healing and transwell assays were performed and analyzed as described in Materials and Methods. (**B** and **D**) Statistical analyse cell migration distances and cell invasion numbers. The data are means ± SD *^#^*P* < 0.05, statistically significant compared with control cell group. (**E**) Representative Western blot images showed that the E-cadherin expression in the cells was increased by treatment of CtBP2-shRNA; meanwhile, the expression of vimentin and MMP-2 were decreased in CtBP2 depletion MDA-MB-231 and MCF-7 cells. However, the expressions were on the contrary in EGFP-CtBP2 cells.

To investigate the molecular mechanisms by which CtBP2 enhances breast cancer cell migration, EGFP-CtBP2 and/or CtBP2-shRNA MDA-MB-231 and MCF-7 clones were established and subjected to wound healing assays. We observed that the track length of EGFP-CtBP2 breast cancer cells migrating into the cell free areas was significantly longer than those of CtBP2-shRNA cells (Figure 6A, 6B). A transwell assay was employed to assess the effect of CtBP2 on cell invasion. We found that the invasion was substantially augmented in breast cancer cells transfected with EGFP-CtBP2 vectors (Figure 6C, 6D). After the transfection of the EGFP-CtBP2 vectors, the expression of the epithelial marker E-cadherin was decreased, while the expression of vimentin was increased. In contrast, CtBP2-shRNA positive cells showed an increased expression of E-cadherin and a decreased expression of vimentin (Figure 6E). We subsequently detected the expression of p16^INK4A^-targeted MMP-2. This expression of MMP-2 was decreased in the cells transfected with CtBP2-shRNA, while MMP-2 was increased in EGFP-CtBP2 positive cells (Figure 6E). These results showed that CtBP2 promoted cell migration and invasion, which correlates with the expression of E-cadherin and p16 ^INK4A^.

### Effect of CtBP2 on tumorigenicity

**Figure 7:**
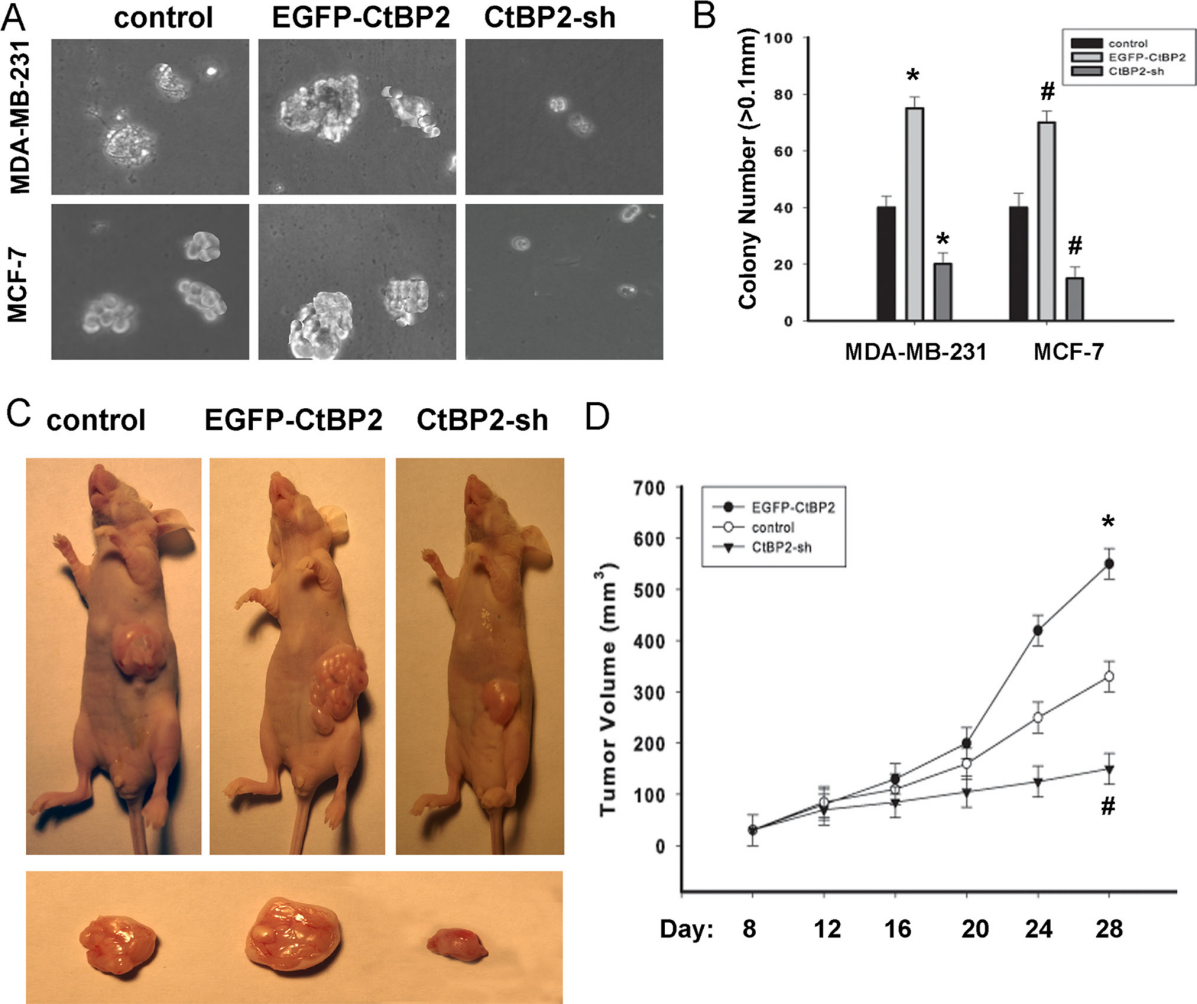
CtBP2 promotes the tumorigenicity of breast cancer cells *in vitro* and *in vivo* (**A**) Anchorage-independent growth assays of CtBP2-overexpressing cells and CtBP2-silenced cells both in MDA-MB-231 and MCF-7 cells. (**B**) The number of colonies with a diameter larger than 0.1 mm was quantified after 10 days of culture. (**C**) Xenograft model in nude mice. MDA-MB-231-CtBP2, MDA-MB-231-CtBP2-shRNA and the respective control cells were inoculated into the nude mice. (**D**) Tumor volumes were measured on the indicated days. Each bar represents the mean ± SD of three independent experiments. *^#^*P* < 0.05, compared with the control group.

As shown in Figures 7A and 7B, EGFP-CtBP2 cells demonstrated a significant increase in the anchorage-independent growth ability in soft agar; however CtBP2-shRNA cells displayed a decrease of the anchorage-independent growth ability in soft agar. Our *in vitro* studies indicated that functional overexpression of CtBP2 makes breast cancer cells phenotypically more malignant, and underexpression of CtBP2 makes the same cells less malignant. Furthermore, we evaluated the effect of CtBP2 on tumorigenicity in nude mice. The tumor growth was measured every 4 days. Similarly, we observed that EGFP-CtBP2 positive tumors grew significantly faster, whereas the tumors formed by CtBP2-shRNA cells grew at a much slower rate (Figure 7C, 7D). The size of tumors formed by CtBP2-shRNA cells was significantly smaller compared with those formed by EGFP-CtBP2 positive cells (*P* < 0.05, Figure 7D).

## DISCUSSION

An increasing body of evidence indicates that CtBP2 is involved in tumorigenesis and tumor progression by the regulation of several essential cellular processes, such as transcriptional repression [9], and is correlated with poor prognosis in a number of tumor types [11, 14, 28–31]. CtBP2 works against important tumor suppressors such as E-cadherin [32], p16^INK4A^ [5], p15^Ink4b^, PTEN, HIPK2 [4], Ink4a/Arf [8] and APC [33], and enhances cell proliferation, migration and invasion. Meanwhile, down-regulation of p16^INK4A^ shows much aggressiveness in breast cancer cells by increasing the expression level of MMP-2 [19]. In ESCC tissues and cells, CtBP2 protein level is elevated by a proteomic study and Guan et al (2013). revealed that CtBP2 contributes to malignant development of ESCC by regulation of p16^INK4A^ [14]. However, the molecular mechanism of CtBP2 in human breast cancer is unclear. Therefore, the present study is aimed to investigate the role and mechanism of CtBP2 in human breast cancer.

Our study shows that the expression of CtBP2 in breast cancer tissues is increased, which was consistent with a previous report by Liu et al. [11]. Meanwhile, p16^INK4A^ expression was decreased in breast cancer tissues. IHC analysis revealed that the expression of CtBP2 in breast cancer samples was positively correlated with breast cancer malignancy. We also found that the increased expression of CtBP2 was a predictor of poor survival. The expression of p16^INK4A^ was significantly lower in breast cancer and was correlated with poor survival in patients with breast cancer. Patients with the combined phenotype of CtBP2 (high) and p16^INK4A^ (low) showed a poorer overall prognosis. This study further showed that CtBP2 negatively regulated the expression of p16^INK4A^, and was positively correlated with cellular proliferation. Thus, these results suggest that CtBP2 might enhance the progression of breast cancer by suppressing the p16^INK4A^ pathway.

This study also showed that CtBP2 is related to histologic grade, metastasis and tumor size, which implies that CtBP2 may be involved in the proliferation and migration of breast cancer. The expression of CtBP2 was detected during the progression of the cell cycle in breast cancer cells. Upon serum stimulation and release from G1, the expression of CtBP2 was clearly increased, concurrent with a decrease of p16^INK4A^ expression. The promotion of growth in the breast cells by wild-type CtBP2 could be explained by the accelerated cell cycle. Down-expression of CtBP2 resulted in suppressed cell growth and an arrest in the cell cycle transition. Similar results were also demonstrated by the colony formation assay and anchorage-independent growth assays. These findings suggest that CtBP2 stimulates cell proliferation and growth in breast cancer. In agreement with previous studies, the present study also found that CtBP2 enhances migration in breast cancer [11]. Genetic manipulation substantiated that CtBP2 promotes migration and invasion by the inhibition of E-cadherin and stimulation of MMP-2. The *in vivo* animal model confirmed that CtBP2 promotes the tumor growth. However, our study has some limitations. While we have confirmed that there are inverse correlations between the expression of CtBP2 and p16 ^INK4A^, the mechanism by which CtBP2 regulates p16 ^INK4A^ expression is still unclear, and follow-up experiments are suggested.

In summary, our results demonstrate that CtBP2 might contribute to the progression of breast cancer by promoting cell proliferation, enhancing cell migration or invasion and inhibiting the expression of p16^INK4A^. A better understanding of the molecular mechanism of CtBP2 in breast cancer development and progression provides novel therapeutic strategies for breast cancer patients.

## MATERIALS AND METHODS

### Specimens

All samples were obtained preoperatively by biopsy. Eighty breast specimens were obtained from January 2000 to December 2003 in the department of pathology, the Affiliated Sixth People's Hospital, Shanghai Jiaotong University. This study was approved by the ethical committee of local institute. Written informed consent was obtained from all patients. The mean post-operative follow-up period for these patients was 78 months (range: 17–129 months). Breast tumors were reviewed regarding histopathologic type based on the WHO classification.

### Immunohistochemistry (IHC) and immunohistochemical analyses

The specimens were fixed in 10% formalin and embedded in paraffin. The procedures were carried out as described as previous methods [11]. Sections were incubated overnight at 4°C with purified CtBP2, p16^INK4A^ and Ki-67 antibodies, which were diluted all at 1:100 with 10 % normal serum in phosphate-buffered saline (PBS). Two pathologists independently scored the results of the staining, and similar results were obtained. CtBP2, p16^INK4A^ and Ki-67 indices were determined as the percentage of all immunostained cells. The mean percentage of CtBP2-positive cells was 61.36%. The samples were considered CtBP2-positive when the percentage of positive cells was > 61.36% and negative when the percentage was ≤ 61.36%. Meanwhile the mean percentage of p16^INK4A^-positive cells was 37.14%, so the samples were considered p16^INK4A^-positive when the percentage of positive cells was > 37.14% and negative when the percentage was ≤ 37.14%.

### Western blot analysis

Western blot was performed similarly to previously described methods [34], using anti-CtBP2 (1:500), anti-p16^INK4A^ (1:1000), anti-PCNA (1:1000), anti-EGFP (1:1000), anti-CDK2 (1:500), anti-CDK4 (1:500), anti-CyclinD (1:500), anti-CyclinE (1:500), anti-p21 (1:1000), anti-Bax (1:1000) anti-E-cadherin (1:1000), anti-vimentin (1:1000), anti-MMP-2 (1:500) and anti-Glyceraldehyde-3-phosphate dehydro-genase (GAPDH) (1:1000, all the above antibodies from Santa Cruz Biotechnology, America). ImageJ (NIH) was used to compare the density of bands on western blot. Mean densitometry data from independent experiments were normalized by GAPDH.

### Cell culture and cell cycle analysis

Breast cell lines HBL-100 and four breast cancer cell lines MDA-MB-415, MDA-MB-231, MDA-MB-453, and MCF-7 (Cell Bank of Type Culture Collection of Chinese Academy of Sciences, Shanghai Institute of Cell Biology, Chinese Academy of Sciences) were cultured in Dulbecco's modified Eagle's medium (DMEM, Invitrogen Life Technologies), supplemented with 10% fetal bovine serum (GibCo BRL, Grand Island, NY), 2 mML-glutamine, 100 units/ml penicillin-G, and 100 mg/ml streptomycin at 37°C and 5% CO_2_. For cell cycle analysis, cells were fixed in 70% ethanol for 1 h at 4°C and then incubated with 1 mg/mL RNase A for 30 min at 37°C. Then, cells were stained with propidium iodide (50 μg/mL PI, Becton–Dickinson, San Jose, CA, USA) in PBS, 0.5% Tween-20, and analyzed using a Becton–Dickinson flow cytometer BD FACScan (Becton–Dickinson).

### Expression plasmid and transfection

The full-length CtBP2 (Genbank Accession No. NM_001083914.1) was isolated from the human cDNA library. The target sequences for CtBP2 gene was 5′- CCCC CTCGAGATGGCCCTTGTGGAT-3′, and 5′-GG GGTAC CTTGCTCGTTGGGGTG-3′, respectively. The PCR fragment was cloned into the pcDNA3.1-EGFP expression vector using the XhoI and Kpn1 restriction sites. The human CtBP2-shRNA expression vector, pSilencer 4.1-CMV, was successfully constructed: 5′-GCGCCTT GGTCAGTAATA-3′, and 3′-CGCGGAACCAGTCATT AT-5′. The non-specific scrambled shRNA with a sequence of 5′-AGCTTCATAAGGCGCATG-3′ and 5′-CATGCG CCTTATGAAGCT-3′ was used as a negative control. Transfection was performed using the Lipofectamine^TM^ 2000 transfection reagent (Invitrogen) according to the manufacturer's protocol.

### Reverse Transcriptase PCR (RT-PCR) and real-time quantitative PCR (RT-qPCR)

Total RNA was prepared from MDA-MB-231 and MCF-7 cells using a Trizol extraction kit according to the manufacturer's procedure. cDNA was synthesized using the Thermo Script RT-PCR system (Invitrogen). Primer pairs for p16^INK4A^ were: sense, 5′-GGGTAGAGGAGG TGCGG-3′ and antisense, 5′-CGGGGATGTCTGAGGGA-3′. The primer pairs for p21 were: sense, 5′-ATGTCAGAA CCGGCTGGGGATGTC-3′, and antisense, 5′-GGGCTT CCTCTTGGAGAAGATC-3′. Cycling conditions were: 95°C for 45 s, 55°C for 45 s, 72°C for 30 s, and a total of 30 cycles. The last cycle was followed by an additional extension step of 72°C for 10 min. GAPDH was used as internal control and was detected using the primers sense, 5′-TGATGACATCAAGAAGGTGGTGAAG-3′ and antisense, 5′-TCCTTGGAGGCCATGTGGGCCAT-3′. Cycling conditions were: 94°C for 30 s, 55°C for 30 s, 72°C for 30 s, and a total of 28 cycles. Densitometric analysis of PCR products was performed with computer software and standardized to the GAPDH product. Quantitative real-time polymerase chain reaction was analyzed by a Lightcycler 480 Detection System (Roche Molecular Biochemicals). RT-qPCR products were detected using SYBR Green. Transcript levels were quantifed by using the 2^ΔCT^ method (ΔCt = Ct_GAPDH_–Ct_Target_).

### Cell proliferation assay

To evaluate the effect of transfection of EGFP-CtBP2 and CtBP2-shRNA, cells were seeded on a 96-well cell culture cluster (Corning Inc., Corning, NY) at a concentration of 2 × 10^4^/well in 100 μL medium and grew overnight. CCK-8 reagents (Dojindo, Kumamoto, Japan) were added to the different subset wells and then incubated at 37°C. The absorbance was quantified using an automated plate reader at a test wave length of 570 nm at different times.

### Colony formation assays

Cells were plated in 60 mm plates (0.5 × 10^3^ cells per plate) and cultured for 10 days. The colonies were stained with 1% crystal violet for 30 s after fixation with 10% formaldehyde for 5 min.

### Wound healing assays

After transfected 48 h, cells were serum starved for 12 h. Then scratching the monolayer with a 10 ml pipette tip, cells were washed with PBS, cultured in 5% FBS-DMEM at 5% CO_2_ and 37°C, and photographed under 20×objective lens every 4 h by inverted Leica phase-contrast microscope (Leica DFC 300 FX).

### *In vitro* invasion assay

A 24-well transwell plate (8 μm pore size, Corning, USA) was used to measure the invasive ability of MDA-MB-231 and MCF-7. Chamber inserts were coated with 200 mg/mL of Matrigel and dried overnight under sterile conditions. Then, 1 × 10^5^ cells were plated in the top chamber. The experiment was carried out for each cell line in triplicates.

### Anchorage-independent growth ability assay

Five hundred cells were trypsinized and suspended in 2 mL complete medium plus 0.3% agar (Sigma, Saint Louis, MI). The agar-cell mixture was planted on top of a bottom layer with 1% agar completed medium mixture. After 10 days, viable colonies that were larger than 0.1 mm were counted. The experiments were repeated at least three times.

### Xenografted tumor model

Female nude mice were housed under standard conditions. The animal protocols were done in agreement with SIBS Guide for the Care and Use of Laboratory Animals and approved by Animal Care and Use Committee, Shanghai Institutes for Biological Sciences. Six-week-old female nude mice were divided into 3 groups (*n* = 8 per group), and the mice were s.c. injected at one site in the left flank with 1 × 10^5^ breast cancer cells. The resulting tumors were measured with calipers every 4 days; length, width, and thickness measurements were obtained with calipers and tumor volumes were calculated. Four weeks after injection, tumors were harvested.

### Statistical analysis

Statistical analyses were performed using SPSS 18.0 software package (SPSS, Inc., Chicago, IL, USA). The association between CtBP2 and p16^INK4A^ expression and clinicopathological features was analyzed using χ^2^ test. CtBP2, p16^INK4A^ and Ki-67 expression was studied using the Spearman rank correlation test. Survival curves were calculated using the Kaplan–Meier method, and the log-rank test was used for analysis. Multivariate analysis was performed using Cox's proportional hazards model. A *P value* < 0.05 was considered as statistical significance.
